# [Ni(OH)_3_W_6_O_18_(OCH_2_)_3_CCH_2_OH]^4–^: the first tris-functionalized Anderson-type heteropolytungstate†
†Electronic supplementary information (ESI) available: Experimental procedures, synthesis details, characterization data, including IR, ESI-MS, TGA, elemental analysis and X-ray crystallography with CIF file. CCDC 1477611. For ESI and crystallographic data in CIF or other electronic format see DOI: 10.1039/c6cc04326g



**DOI:** 10.1039/c6cc04326g

**Published:** 2016-06-15

**Authors:** Nadiia I. Gumerova, Alexander Roller, Annette Rompel

**Affiliations:** a Universität Wien , Fakultät für Chemie , Institut für Biophysikalische Chemie , Althanstr. 14 , 1090 Wien , Austria . Email: annette.rompel@univie.ac.at ; www.bpc.univie.ac.at; b Universität Wien , Fakultät für Chemie , Institut für Anorganische Chemie , Währinger Str. 42 , 1090 Wien , Austria

## Abstract

Na_2_[TMA]_2_[Ni(OH)_3_W_6_O_18_(OCH_2_)_3_CCH_2_OH]·9H_2_O represents the first covalent tris-functionalized Anderson-type heteropolytungstate and was characterized by single-crystal X-ray diffraction, electrospray ionization mass spectrometry, TGA and IR spectroscopy. Zeta potential measurements in solutions containing human serum albumin were performed to investigate electrostatic interactions with [Ni(OH)_3_W_6_O_18_(OCH_2_)_3_CCH_2_OH]^4–^.

## Abstract 2

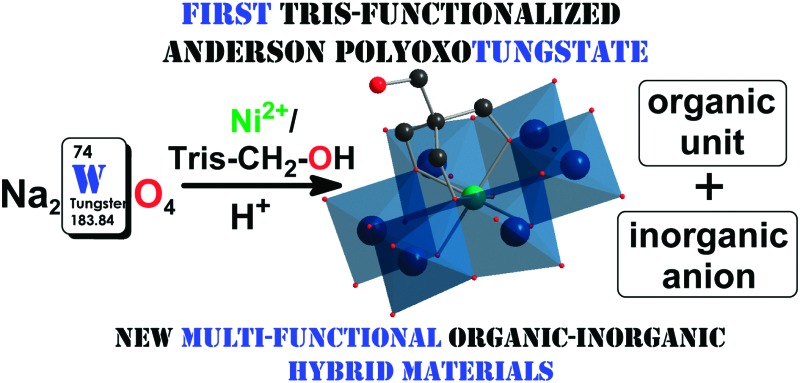
Na_2_[TMA]_2_[Ni(OH)_3_W_6_O_18_(OCH_2_)_3_CCH_2_OH]·9H_2_O represents the first covalent tris-functionalized Anderson-type heteropolytungstate and was characterized by single-crystal X-ray diffraction, electrospray ionization mass spectrometry, TGA and IR spectroscopy.

Polyoxometalates (POMs) are an exceptional brand of coordination compounds consisting of early transition metal atoms linked by shared oxygen atoms.^1^ Due to their wide range of size, structure and composition, they possess unique thermal, redox, magnetic, optical and bioactive properties and exhibit an enormous potential for application in various fields.^2^ Especially Lindqvist- and Keggin-type POMs have been shown to exhibit biological activity *in vitro* as well as *in vivo* ranging from anti-cancer, antibiotic, and antiviral to antidiabetic effects.^3^ The design of organically functionalized (inorganic) POM hybrids *via* a controllable synthesis has gained much attention since the combination of organic and inorganic components into one single compound provides new properties that benefit from the strengths of both units for multi-functional hybrid materials.^4^

From the first by Knoth^5^ reported formation of O-alkylated anions, [PM_12_O_39_(OMe)]^2–^ (M = Mo, W), the direct covalent functionalization with tris-alkoxo organic ligands (tris(hydroxymethyl)methane (RC(CH_2_OH)_3_)) has been widely applied for different POM archetypes.^6^ The controllable synthesis of functionalized Anderson-type polyoxomolybdates (POMo) has gained much attention^7^ since the first Anderson-type hybrid structure was described by Hasenknopf in 2002.^7*a*^ This functionalization is achieved by replacing three or six protons of the B-type Anderson-structure, which are attached to the μ_3_-O or even to the less basic μ_2_-O atoms, with organic tris-ligands. Single- or double-side grafted δ-, χ- or ψ-isomers of functionalized Anderson-type POMos, [M(OH)_6_Mo_6_O_18_]^*n*–^ (M = Zn^2+^, Ni^2+^, Cr^3+^, Mn^3+^, Al^3+^, Fe^3+^, Ga^3+^), can be obtained *via* the re-arrangement of the octamolybdate-anion or by applying a pre-synthesized Anderson polyanion under different reaction conditions.^7,8^ However, to the best of our knowledge nobody succeeded so far in obtaining organically functionalized Anderson-type polyoxotungstates (POTs). The derivatization of POTs applying tripodal ligands is only known for mixed anions exhibiting two addenda atoms.^6^ It should be noted that in these structures the anchoring of organic groups is only observed onto the V_3_O_13_ fragments of the Dawson or Keggin anions or onto the Ni_6_-core in Ni_6_PW_9_.^9^ The disinclination to easily form W–O–C bonds can be explained by the inertness of the bridging oxygen atoms in the WO_6_ octahedra. The covalent functionalization of lacunary POTs is accomplished by the formation of W–O–X bonds (X = N, Si, P, As, Sn) with the more nucleophilic terminal O atoms at the surface of the lacuna.^10^

The application of [TeW_6_O_24_]^6–^ (TEW, A-type Anderson-POT) has recently been expanded to its successful use as an additive in protein crystallization.^11^ Here, [TeW_6_O_24_]^6–^ demonstrated superiority over other POM archetypes owing to its good water solubility, pH-stability, disk-shape structure and relatively high negative charge. In the presence of proteins [TeW_6_O_24_]^6–^ exhibits no proteolytic activity,^11*d*^ does not alter the protein structure but provides useful anomalous signal for phasing due to the high molar weight of tungsten. So far and uniquely observed, [TeW_6_O_24_]^6–^ mediates crystal contacts^11b–e^ between protein molecule favoring crystal growth. Some protein crystals that were co-crystallized with [TeW_6_O_24_]^6–^ possess a higher resolution than the crystals obtained without [TeW_6_O_24_]^6–^.^11*e*^ There is an urgent need for covalently functionalized Anderson-type POTs to enable specific binding options *via* organic functional groups between proteins and the Anderson-type POT. Therefore, we developed a method to prepare a tris-functionalized Anderson POT. Herein, we report the first synthesis of a tris-functionalized Anderson-type polyoxotungstate-anion, [Ni(OH)_3_W_6_O_18_(OCH_2_)_3_CCH_2_OH]^4–^ (**NiW_6_-Tris-CH_2_OH**), and its characterization by single-crystal X-ray analysis, electrospray ionization mass spectrometry (ESI-MS), IR as well as TGA. Zeta potential measurements were performed to investigate and compare the electrostatic interactions of **NiW_6_-Tris-CH_2_OH**, with its pure inorganic counterparts [Ni(OH)_6_W_6_O_18_]^4–^, [Cr(OH)_3_W_6_O_21_]^6–^ and [TeW_6_O_24_]^6–^ on human serum albumin HSA.

The full protonation of the six μ_3_-O atoms (forming the coordination sphere of the heteroatom) is only present in B-type Anderson anions^1^ and so far exclusively observed for [Ni(OH)_6_W_6_O_18_]^4–^.^12^ Due to this fact, [Ni(OH)_6_W_6_O_18_]^4–^ has been chosen as the starting compound for the tris-functionalization. When choosing between different tris(hydroxymethyl)methane ligands, we took into consideration a previous report^7*b*^ showing the preference of Ni^2+^ for the amino functionality of the Tris-NH_2_ by forming Ni–NH_2_ complexes and no Tris-NH_2_-functionalized Ni-centered Anderson POMo product. Therefore, no amino-containing tris ligand was used, but pentaerythritol CH_2_OH–C(CH_2_OH)_3_ (Tris-CH_2_OH) was chosen as the ligand for grafting onto the Ni-containing POT [Ni(OH)_6_W_6_O_18_]^4–^. By applying Tris-CH_2_OH one OH-group still stays available after anchoring and can be used for further post-functionalization by esterification with acid anhydrides^13^ making Tris-CH_2_OH the ligand of choice.

**NiW_6_-Tris-CH_2_OH** is assembled by a condensation reaction of Tris-CH_2_–OH with the [Ni(OH)_6_W_6_O_18_]^4–^ anion in an acidified aqueous solution (Scheme 1). Initially, an aqueous solution of Ni^2+^–WO_4_^2–^–H^+^ with a molar ratio of 1 : 6 : 6 (Ni : W : H^+^) at pH 6.5 was prepared. The reaction mixture was kept 10 days at ambient conditions in order to increase the concentration of [Ni(OH)_6_W_6_O_18_]^4–^. The pH of the obtained blue solution was reduced to 3.5 with diluted nitric acid. Tris-CH_2_OH was added in fivefold excess before refluxing the solution for 5 h followed by the addition of tetramethylammonium (TMA) chloride as counter ions. Providing less excess of the tripodal ligand leads to no functionalization, however, a higher excess of Tris-CH_2_OH did not lead to the double sided functionalized anion and solely the one-side functionalized anion was obtained.

**Scheme 1:**
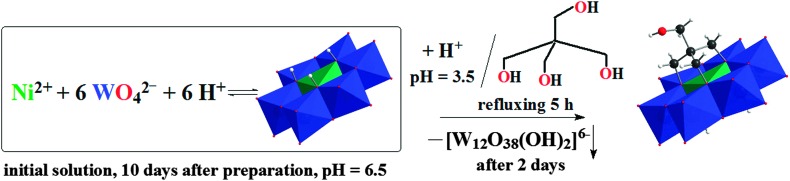
The synthesis of **NiW_6_-Tris-CH_2_OH**; color code: WO_6_, blue octahedra; NiO_6_, green octahedron; O, red; C, black; H, white.

Some competing processes can take place in the reaction mixture: (1) self-assembly of **NiW_6_-Tris-CH_2_OH** from monomeric anions, (2) formation of **NiW_6_-Tris-CH_2_OH** by grafting tripodal ligand onto formed [Ni(OH)_6_W_6_O_18_]^4–^ and (3) partial formation of isopolyparticles as byproducts due to the influence of the low pH. After filtration of the reaction solution a salt has been isolated and identified by single-crystal X-ray diffraction measurements and IR spectroscopy as metatungstate TMA_6_[W_12_O_38_(OH)_2_] (see Fig. S2, ESI†). From the blue mother liquor the single-side tris-functionalized Anderson-type δ isomer [Ni(OH)_3_W_6_O_18_(OCH_2_)_3_CCH_2_OH]^4–^ (see Fig. 1) has been obtained in a quite good yield of 55%. The main advantage of this synthesis route is the possibility to straightforwardly obtain the functionalized Anderson-type cluster in a one-pot reaction which paves the way for more Ni-Anderson tungstate hybrids by applying other tripodal ligands or exploiting the reactivity of the free OH-group in Tris-CH_2_OH.

**Fig. 1:**
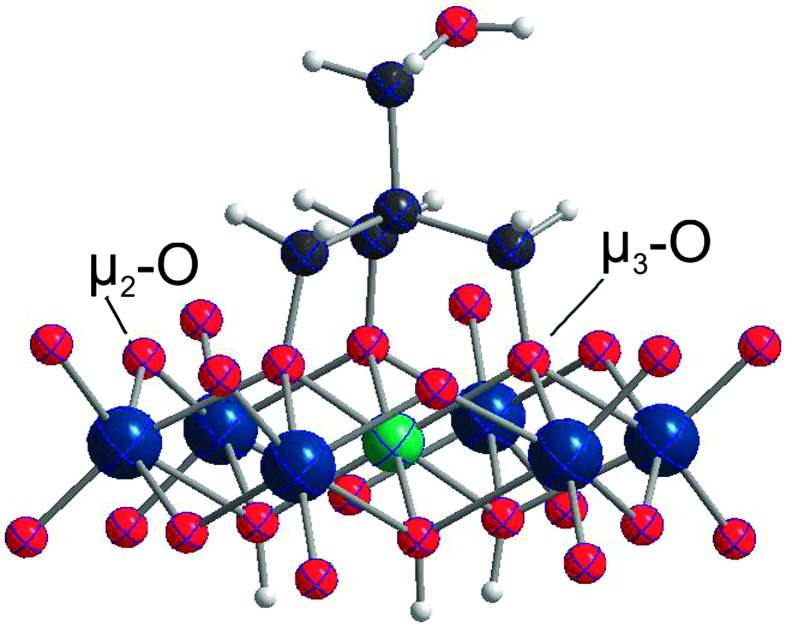
ORTEP drawing of **NiW_6_-Tris-CH_2_OH** (50% probability ellipsoid, H atoms represented in ball mode): W, blue; Ni, green; O, red; C, black; H, light grey.

Our attempts to carry out the synthesis without addition of extra acid to the initial solution of Ni^2+^–WO_4_^2–^–H^+^ or to an aqueous solution of Na_4_[Ni(OH)_6_W_6_O_18_] (pH = 6.5) that had been refluxed 3, 5 or 12 h applying various amounts of Tris-CH_2_OH were not successful. In all cases the crystallization of a non tris-decorated product was observed.

The impact of the pH during the Tris-POMo synthesis of [Cr(OH)_6_Mo_6_O_18_]^4–^ and [Mn(OH)_6_Mo_6_O_18_]^4–^, leading to differently formed isomers has been very recently investigated and discussed by the Wei group.^7g,h^ They showed that the addition of protons to the reaction mixture during the functionalization leads to the protonation of one of the μ_2_-O atoms in the Anderson anion. This protonated μ_2_-O represents a highly reactive site where the tris-ligand is anchored on two μ_3_-O atoms and one μ_2_-O atom forming the χ isomer. However, in the case of [Ni(OH)_6_W_6_O_18_]^4–^, the presence of extra protons does not lead to the formation of the χ isomer but to the δ isomer, **NiW_6_-Tris-CH_2_OH**, which is most likely caused by the inertness of the μ_2_-O atoms inhibiting the formation of a W–O–C bond with alkoxo ligands from the W–O–W fragment.

Na_2_[TMA]_2_[Ni(OH)_3_W_6_O_18_(OCH_2_)_3_CCH_2_OH]·9H_2_O crystallizes in the triclinic space group *P*1. The structure is composed of a [Ni(OH)_3_W_6_O_18_(OCH_2_)_3_CCH_2_OH]^4–^ anion, two tetramethyl-ammonium and two sodium cations. **NiW_6_-Tris-CH_2_OH** shows the common Anderson heteropolyanion structure which consists of six WO_6_ octahedra arranged hexagonally around the central {NiO_6_} octahedron (Fig. 1). The ligand replaces three protons from the Ni(OH)_6_ core in order to attach to the disk shape anion. The Ni–O bond lengths are in the range from 2.018(11) to 2.042(15) Å and the μ_3_-O–C bond lengths are in the range from 1.373(2) to 1.389(2) Å, which is in good agreement with the corresponding bond lengths of the Anderson-anion [H_2_NiMo_6_O_18_{(OCH_2_)_3_CCH_2_OH}_2_]^2–^.^7*a*^ The structure analysis indicates that the lengths of all three types of W–O bonds (W–μ_2_-O, W–μ_3_-O, W=O_terminal_) are also close to the one of the parent species [Ni(OH)_6_W_6_O_18_]^4–^ and to the corresponding POMo analogues (Table 1).

**Table 1:** Selected bond lengths in functionalized and non-functionalized Ni-containing Anderson POMs

	[Ni(OH)_6_W_6_O_18_]^4– ^ ^11^	[Ni(OH)_3_W_6_O_18_(OCH_2_)_3_CCH_2_OH]^4–^	[Ni(OH)_6_Mo_6_O_18_]^4– ^ ^14^	[H_2_NiMo_6_O_18_{(OCH_2_)_3_CCH_2_OH}_2_]^2– ^ ^7*a*^
Distances, Å:				
M–μ_2_-O	2.229(11)–2.243(12)	2.227(10)–2.258(12)	2.223(9)–2.238(9)	2.078(8)–2.317(10)
M–μ_3_-O	1.915(10)–1.958(11)	1.912(10)–1.946(11)	1.911(12)–1.972(13)	1.861(13)–1.964(12)
M–O_terminal_	1.723(13)–1.744(11)	1.736(11)–1.742(10)	1.708(11)–1.726(12)	1.695(12)–1.708(10)
Ni–μ_3_-O	2.027(4)–2.051(3)	2.018(4)–2.042(3)	2.024(8)–2.050(7)	2.010(2)–2.085(2)
μ_3_(μ_2_)-O–C	—	1.373(2)–1.389(2)	—	1.430(3)–1.453(2)

The **NiW_6_-Tris-CH_2_OH** anion interacts with sodium counter cations *via* their W=O terminal oxygen atoms (O7, O11), hence the coordination sphere of Na^+^ consists of two oxygen atoms from two neighboring polyanions and three water molecules exhibiting trigonale bipyramide geometry (see Fig. S1, ESI†). That way, alternate heteropolyanions and sodium NaO_5_ polyhedra form parallel chains interacting through intermolecular hydrogen bonds. Crystal lattice water molecules, the terminal oxygen atoms in the anion, the OH functionality in the grafted organic ligands and the three protons found on the undecorated side form a three dimensional system of H-bonds in the crystal structure (see Fig. S1, ESI†).

IR spectroscopy was applied to investigate the anion **NiW_6_-Tris-CH_2_OH** (see Fig. S3, ESI†). The characteristic peaks of the core structure are all in agreement with the peaks observed in the spectrum of Na_4_[Ni(OH)_6_W_6_O_18_]·16H_2_O.^12^ The stretching vibrations of the terminal W=O units appear at 955 and 940 cm^–1^. The peaks at 884 cm^–1^ and in the region of 470–750 cm^–1^ correspond to the antisymmetric and symmetric deformation vibrations of W–O–W and W–O–Ni bridging fragments. The re-duction of symmetry from *D*_3d_ in [Ni(OH)_6_W_6_O_18_]^4–^ to *C*_2h_ in **NiW_6_-Tris-CH_2_OH** leads to the reduction in the intensity of the W–O–W vibrations bands at 560 and 725 cm^–1^. The three peaks appearing at 1105, 1072 and 1035 cm^–1^ could be assigned to C–O stretching vibrations, indicating the successful grafting of Tris-CH_2_OH.

Evidence for the existence of an intact **NiW_6_-Tris-CH_2_OH** polyanion in solution and its exact stoichiometric composition was obtained from ESI-MS. The ESI-MS spectrum of **NiW_6_-Tris-CH_2_OH** demonstrates its complex character and exhibits a peaks envelope at *m*/*z* = 828.8 and *m*/*z* = 839.9 which can be unambiguously assigned to the double charged anions Na_2–*x*_H_*x*_[Ni(OH)_3_W_6_O_18_(OCH_2_)_3_CCH_2_OH]^2–^ (*x* = 0, 1; calculated mass of 828.8 and 839.9) indicating the presence of the intact one-side grafted cluster in the solution. A series of peak envelopes in the rest of the spectra could be assigned to different common POTs fragments (HWO_4_^–^, W_2_O_7_^2–^, HW_2_O_7_^–^, NaW_2_O_7_^–^, W_3_O_10_^2–^, HW_3_O_10_^2–^ *etc.*) and some specific decomposition products, *e.g.* NaNiW_4_O_13_(OH)_3_^–^, Na_2_H_3_[Ni(OH)_3_W_3_O_10_(OCH_2_)_3_CCH_2_OH]^–^, NaH[Ni(OH)_3_W_5_O_14_(OCH_2_)_3_CCH_2_OH]^–^ *etc.* (see Fig. S4 and S5, ESI†).

The TGA data were used to examine the weight loss and thermal stabilities of the synthesized hybrid POT. The TG curve shows three weight-loss regions up to 600 °C due to dehydration followed by disintegration of the various organic complexes (see Fig. S6, ESI†). The dehydration takes place in two stages, at 33–160 °C and 160–230 °C, indicating the presence of differently strong coordinated water in the structure. The loss of three OH-groups from the coordination sphere of the of Ni^2+^ could not be found as a separate step on the TG curve and presumably takes place together with the loss of the organic ligand between 230 and 530 °C.

To investigate the interactions between the negatively charged Anderson-tungstate anions and the positively charged surface regions in human serum albumin (HSA), zeta potential measurements on a series of POTs with Ni, Cr and Te as heteroatom ([Ni(OH)_6_W_6_O_18_]^4–^, [Cr(OH)_3_W_6_O_21_]^6–^, [TeW_6_O_24_]^6–^) and the tris-functionalized product **NiW_6_-Tris-CH_2_OH** were applied. The zeta potential was measured in NaOAc buffered (50 mM, pH 4.0) solutions containing 1 mg mL^–1^ (0.015 mM) of HSA. The concentration of heteropolyanions {XW_6_} was varied from 0 to 1 mM and all solutions were incubated overnight at 4 °C prior to the measurements. The molar ratio between protein and {XW_6_} anions varied from 1 : 0 to 1 : 67. The results are illustrated in Fig. 2 and indicate a progressive decrease of the zeta potential of the surface of HSA with increasing POT concentration for all compounds. The charge inversions took place at the following concentrations: 0.25 mM for [TeW_6_O_24_]^6–^, 0.3 mM for [Cr(OH)_3_W_6_O_21_]^6–^, 0.3 mM for **NiW_6_-Tris-CH_2_OH** and 0.35 mM for [Ni(OH)_6_W_6_O_18_]^4–^. The slightly lower concentration of **NiW_6_-Tris-CH_2_OH** for charge inversion in comparison to that of [Ni(OH)_6_W_6_O_18_]^4–^ may be caused by additional interaction between the OH functionality and the protein. The interactions of triply charged Anderson-type POMos with bovine serum albumin also showed charge inversions of BSA at low concentrations of anions decorated with Tris-CH_2_OH bearing a high negative surface charge.^7*f*^

**Fig. 2:**
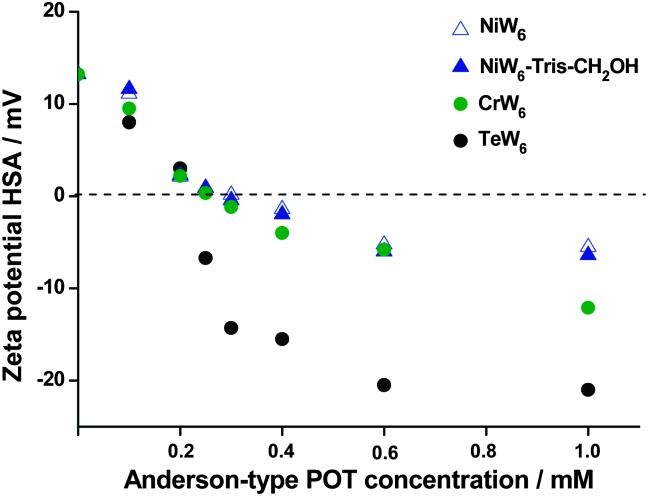
The zeta potential of HSA (NaOAc buffered solution, 50 mM, pH 4.0 with concentration 1 mg mL^–1^ (0.015 mM)) as a function of Anderson POT concentration; NiW_6_ – [Ni(OH)_6_W_6_O_18_]^4–^; **NiW_6_-Tris-CH_2_OH** – [Ni(OH)_3_W_6_O_18_(OCH_2_)_3_CCH_2_OH]^4–^; CrW_6_ – [Cr(OH)_3_W_6_O_21_]^6–^; TeW_6_ – [TeW_6_O_24_]^6–^.

In summary, 14 years after the first successful tris functionalization of an Anderson type POMo, **NiW_6_-Tris-CH_2_OH** is the first example of a covalently modified Anderson-type heteropolytungstate. The hydrated sodium tetramethylammonium salt of **NiW_6_-Tris-CH_2_OH** has been synthesized in good yields and has been extensively characterized in solid state and in solution. Zeta potential measurements performed on [Ni(OH)_3_W_6_O_18_(OCH_2_)_3_CCH_2_OH]^4–^ in the presence of HSA demonstrated its applicability to induce charge inversion. The introduction of tris-ligand to Anderson-type POTs suggests existence of a rich tris-functionalized POT chemistry that will be elucidated in the future. The proposed synthesis strategy opens a route for new multi-functional organic–inorganic hybrid materials for various applications.

The research was funded by the Austrian Science Fund (FWF): P27534. N. I. G. thanks OEAD for a scholarship of the Scholarship Foundation of the Republic of Austria for Postdocs. The authors are grateful to Ing. P. Unteregger for support with the ESI-MS measurements at the MS Centre, Univ. of Vienna. We thank Ao.Uni.-Prof. E. Libowitzky for access to ATR-IR measurements and Ao.Uni.-Prof. C. L. Lengauer for TGA measurements at the Institut für Mineralogie und Kristallographie, Univ. of Vienna. Special thanks to Ao.Univ.-Prof. F. Gabor and Mag. J. Gausterer from the Dep. of Pharmaceutical Technology and Biopharmaceutics, Univ. of Vienna for access to zeta potential measurements. Lastly, the authors wish to thank T. Caldera-Fraile, MSc. for the synthesis of CrW_6_ and Dr A. Bijelic, Dipl.-Chem. C. Molitor, A. Blazevic, MSc. and E. Al-Sayed, MSc. for valuable discussions regarding this work.
