# Supplementary material for: [Ni(OH)3W6O18(OCH2)3CCH2OH]4–: the first tris-functionalized Anderson-type heteropolytungstate
Source: Chem Commun (Camb). 2016 Jun 15;52(59):9263–6. doi: 10.1039/c6cc04326g (PMC5040144; doi:10.1039/c6cc04326g)
Supplement: Supplementary file 1 [file CC-052-C6CC04326G-s001.pdf]

Electronic Supplementary Material (ESI) for Chemical Communications.
This journal is © The Royal Society of Chemistry 2016

ELECTRONIC SUPPLEMENTARY INFORMATION

$[Ni(OH)_3W_6O_{18}(OCH_2)_3CCH_2OH]^{4-}$: THE FIRST TRIS-FUNCTIONALIZED ANDERSON-TYPE HETEROPOLYTUNGSTATE

Nadiia I. Gumerova, ‡[a] Alexander Roller[b] and Annette Rompel*[a]

[a] Universität Wien, Fakultät für Chemie, Institut für Biophysikalische Chemie, Althanstr. 14, 1090 Wien, Austria. www.bpc. univie.ac.at. E-Mail: annette.rompel@univie.ac.at

‡ On leave from: Department of Inorganic Chemistry, Donetsk National University, 21021 Vinnytsia, Ukraine. E-Mail: n.gumerova@donnu.edu.ua

[b] Universität Wien, Fakultät für Chemie, Institut für Anorganische Chemie, Währinger Str. 42, 1090 Wien, Austria

Content

1. General methods and materials. 3
2. Synthesis of $Na_2[TMA]_2[NiW_6O_{18}(OH)_3(OCH_2)_3CCH_2OH]\cdot 9H_2O$. 3
3. X-ray crystallography. 4
4. IR spectroscopy. 7
5. Mass spectrometry measurements 8
6. Thermogravimetric analysis. 9
References 9

1. General methods and materials.

The reagents were reagent grade and used as purchased without further purification. $Na_4[Ni(OH)_6W_6O_{18}]\cdot 16H_2O$ and $Na_6[TeW_6O_{24}]\cdot 22H_2O$ were obtained according to the reported procedures[1,2] and its identity was confirmed by IR spectroscopy.

Attenuated total reflection Fourier−transform Infrared Spectroscopy: IR spectra were recorded on a Bruker Tensor 27 IR Spectrometer equipped with a single−reflection diamond−ATR unit. **Elemental Analysis:** The determination of C/H/N/O was done using an "EA 1108 CHNS-O" elemental analyzer by Carlo Erba Instruments. **Thermogravimetric analysis:** TGA was performed on a Mettler SDTA851e Thermogravimetric Analyzer under nitrogen flow with a heating rate of 5 K min^{-1} in the region 298−1023 K. **Electrospray Ionization Mass Spectrometry:** The title compound was investigated with an ESI−Qq−oaRTOF supplied by Bruker Daltonics Ltd. Bruker Daltonics Data Analysis software was used to analyze the results. The measurement was carried out in a 1:1 mixture of water/MeCN, collected in negative ion mode and with the spectrometer calibrated with the standard tune−mix to give a accuracy of *ca.* 5 ppm in the region of *m/z* 300−3000. **Zeta potential:** For the zeta potential measurements a Zetasizer Nano ZS (Malvern, United Kingdom) was used with a measurement position of 2 mm. The zeta potential was measured in NaOAc buffered (50 mM, pH 4.0) solutions containing 1 $mg\cdot mL^{-1}$ of HSA. Every solution was measured 30 times at a temperature of 25°C and the mean value was used as final data. The pH of every solution was measured after the experiment, and no significant pH variation was noted (3.91 – 4.11).

2. Synthesis of $Na_2[TMA]_2[NiW_6O_{18}(OH)_3(OCH_2)_3CCH_2OH]\cdot 9H_2O$.

A sample of $Na_2WO_4\cdot 2H_2O$ (0.83 g, 2.5 mmol) was dissolved in water (20 mL) and 2.5 mL of HNO_3 (1.0 M) was added. $Ni(NO_3)_2\cdot 2H_2O$ (0.12 g, 0.4 mmol) was dissolved in water (5 mL) and added dropwise to the acidified solution of Na_2WO_4. The molar ratio of $Ni^{2+}/WO_4^{2-}/H^+$ in an aqueous solution is 1:6:6 (Ni/W/H^+) and corresponds to the maximum amount of $[Ni(OH)_6W_6O_{18}]^{4-}$ (0.42 mmol) according to the chemical equation: $Ni^{2+} + 6\ WO_4^{2-} + 6\ H^+ \leftrightarrows [Ni(OH)_6W_6O_{18}]^{4-}$. The reaction mixture of green color was kept at room temperature for 10 days. The color of the reaction mixture changed from green

to blue (pH 6.5) and the solution´s pH was adjusted to pH 3.5 with diluted HNO_3 and heated to reflux followed by addition of pentaerythritol (0.27 g, 2 mmol). After refluxing at 80°C for 5 hours TMA−Cl was added (0.55 g, 5 mmol) and the solution was cooled down to room temperature. Colorless crystals of $[TMA]_6[W_{12}O_{38}(OH)_2]\cdot nH_2O$ appeared within 2 days with the following cell parameters a = 12.9872(5), b = 21.0354(9), c = 13.1053(5), α = γ = 90°, β = 91.6841(2). Evaporation of the blue filtrate in air led to formation of blue block-shaped crystals of the title compound within 2 to 3 weeks. Yield: 0.45 g (55 % based on W). Elemental analysis: found for $C_{13}H_{54}N_2Na_2NiO_{34}W_6$ (calculated): C 7.95 (7.84); H 2.85 (2.73); N 1.44 (1.42); O 27.39 (27.34) %. IR (cm^{-1}): 3463 (m), 3363 (s), 3220 (s, br), 1647 (m), 1620 (m), 1483 (s, sh), 1448, 1415 (w), 1396 (w), 1332 (w), 1105 (m), 1072 (w), 1035 (w), 1001 (w), 943 (s), 923 (s), 876 (s), 646 (s), 582 (m), 550 (m), 489 (w).

Synthesis of $Na_6[Cr(OH)_3W_6O_{21}]\cdot 22H_2O$. 30 mL of an aqueous solution containing $Na_2WO_4\cdot 2H_2O$ (3.3 g, 10 mmol) was heated at approximately 80 °C, followed by the addition of 0.1 g of boric acid. The final pH of the solution was adjusted to 7 with diluted HCl. $CrCl_3\cdot 6H_2O$ (0.54 g, 2 mmol) dissolved in 2 mL water was added dropwise. A light turbidity occurred and it was waited until the solution cleared again before adding the next drop. After the complete addition of the $CrCl_3\cdot$6H2O, the solution remained turbid. The final pH of 8 was adjusted by addition of diluted NaOH. The solution was stirred and heated at approximately 80 °C for half an hour. Afterwards, it was cooled down to room temperature, centrifuged to remove unreacted educts and transferred to crystallization beakers. After several weeks, green single crystals were obtained.

3. X-ray crystallography.

The X-ray data were measured on a Bruker D8 Venture equipped with multilayer monochromator, MoKα INCOATEC micro focus sealed tube and Kryoflex cooling device. The structure was solved by direct methods and refined by full-matrix least-squares techniques. Non-hydrogen atoms were refined with anisotropic displacement parameters. Hydrogen atoms were inserted at calculated positions and refined with riding coordinates. The following software was used for the structure solving procedure:

Frame integration, Bruker SAINT software package[3] using a narrow-frame algorithm (absorption correction), SADABS[4] (structure solution), SHELXS-2013[5] (refinement), SHELXL-2013[5], OLEX2[6], SHELXLE[7] (molecular diagrams), OLEX2[6]. Experimental data and CCDC-code can be found in Table 1. Crystal packing is displayed in Figure S1.

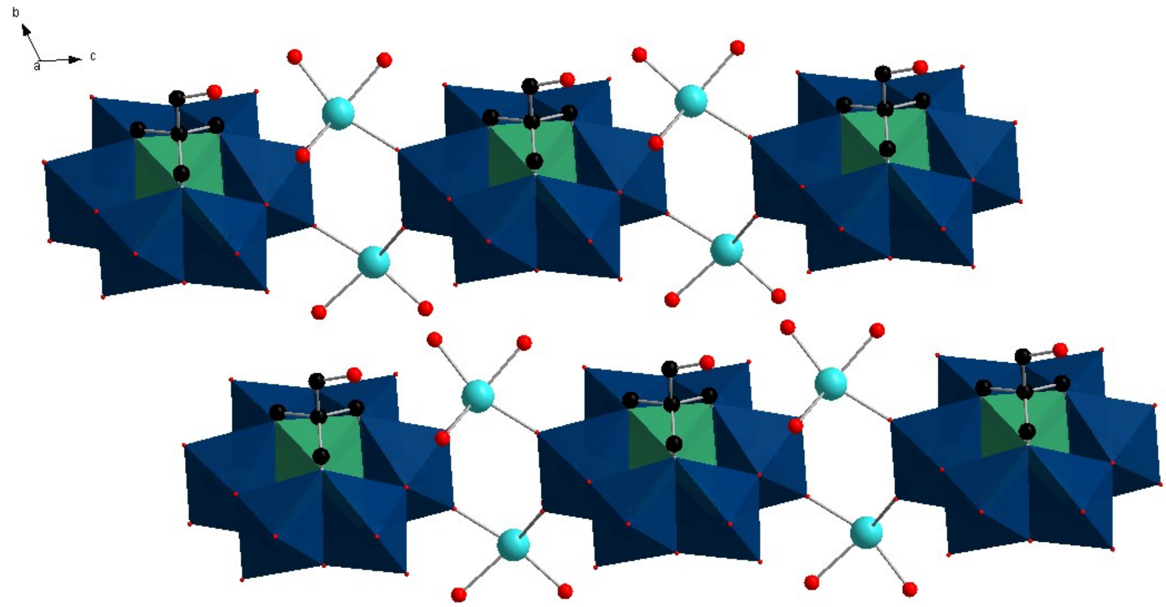

Fig. S1. Crystal packing arrangement in **$Na_2[TMA]_2[NiW_6O_{18}(OH)_3(OCH_2)_3CCH_2OH]\cdot 9H_2O$** along the a-plane. Counter cations, H_2O and H are omitted for clarity.

Table S1. Crystallographic data.

	$Na_2[TMA]_2[NiW_6O_{18}(OH)_3(OCH_2)_3CCH_2OH]\cdot 9H_2O$
Empirical formula	$C_{13}H_{54}N_2Na_2NiO_{34}W_6$
CCDC-Code	1477611
Formula weight M_r	1990.37
Crystal system	Triclinic
Space group	P–1
T, K	100
a, b, c (Å)	9.6825(4)
	10.3943(5)
	11.7580(6)
α, β, γ (°)	113.7330(12)°
	102.5434(12)°
	90.4790(12)°
V (Å^3)	1051.34(9)
Z	1
D_{calc}, g/cm^3	3.144
μ, mm^{-1}	16.905
Abs. correction type	Multiscan
Abs. correction Tmin	0.5679
Abs. correction Tmax	0.746
F(000)	912.0
Crystal size, mm	0.158 × 0.153 × 0.096
Theta range for data collection	2.94° – 25.34
Index ranges	$-11 \le h \le 11$
	$-12 \le k \le 12$
	$-14 \le l \le 14$
Reflections collected	46417
Independent reflections	3820
Rint	0.0365
Data / restraints / parameters	3820/22/319
Goodness-of-fit on F^2	1.32
Final *R* indices	R_F = 0.0179, wR^2 = 0.0457
	(all data)
	R_F = 0.0179, wR^2 = 0.0457
	(I>2σ(I))
Largest diff. peak and hole, e. Å^{-3}	0.83/-1.91

4. IR spectroscopy.

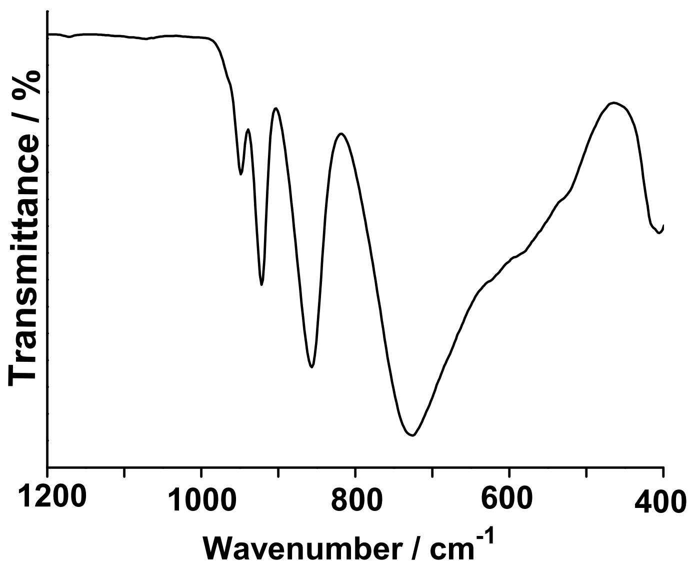

Fig. S2. IR spectra of $[TMA]_6[W_{12}O_{38}(OH)_2]\cdot nH_2O$.

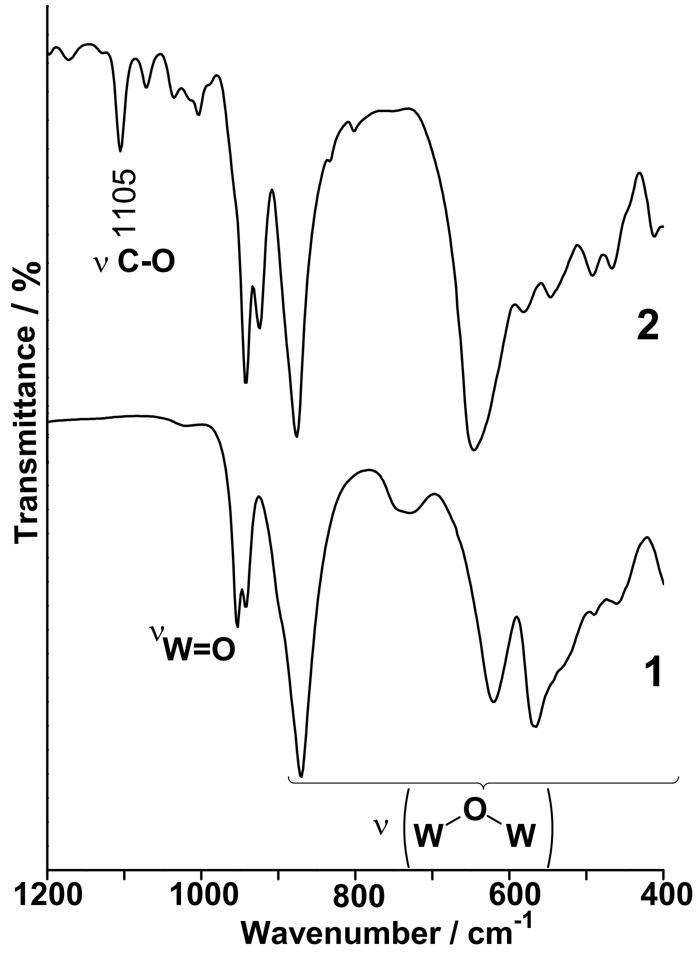

Fig. S3. IR spectra of compounds $Na_4[Ni(OH)_6W_6O_{18}]\cdot 16H_2O$ (1) and $Na_2[TMA]_2[Ni(OH)_3W_6O_{18}(OCH_2)_3CCH_2OH]\cdot 9H_2O$ (2).

5. Mass spectrometry measurements

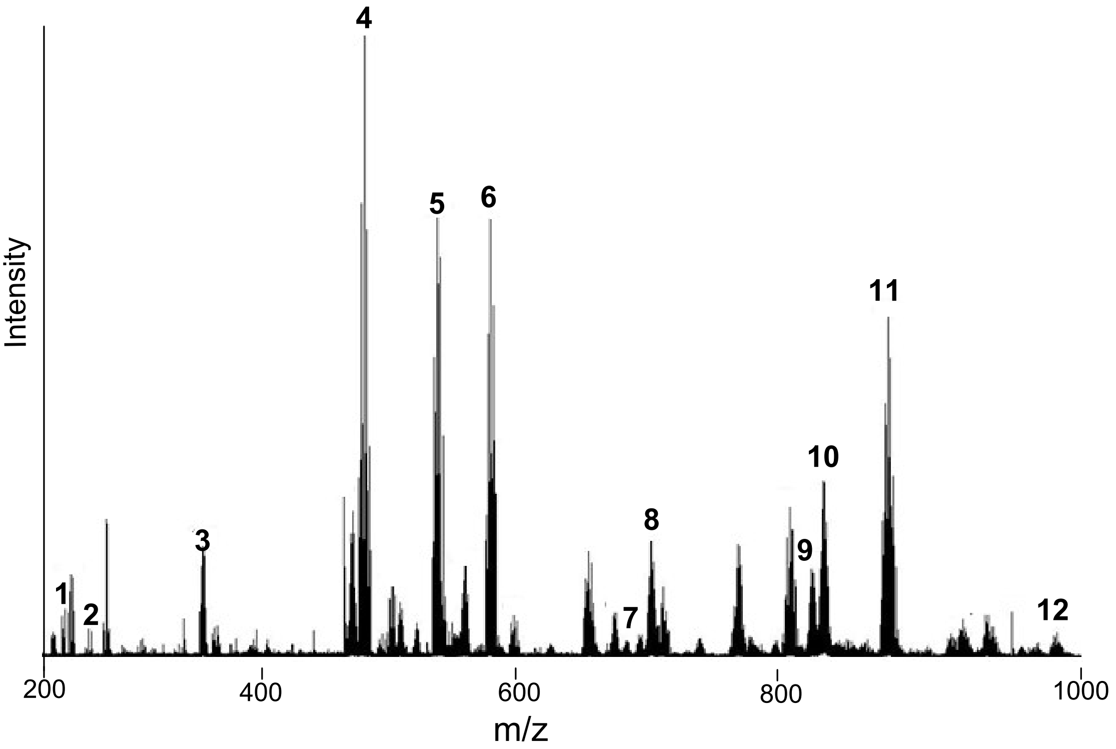

Fig. S4. Negative ion-mode ESI-MS spectrum of $Na_2[TMA]_2[Ni(OH)_3W_6O_{18}(OCH_2)_3CCH_2OH]\cdot9H_2O$ in mixed H_2O/CH_3CN.

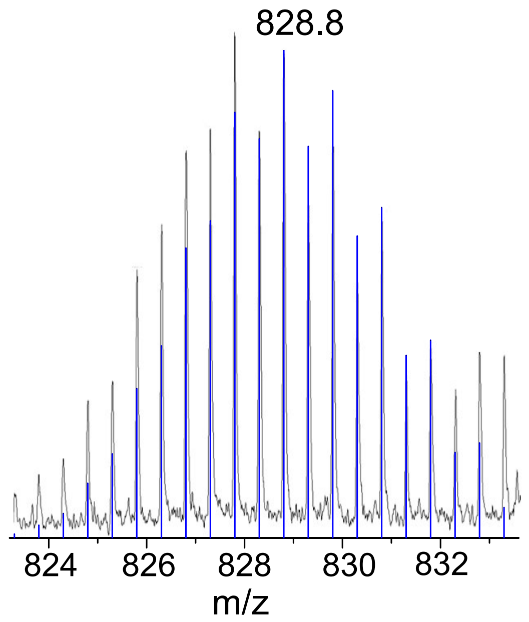

Fig. S5. ESI-MS peak envelope of $NaH[Ni(OH)_3W_6O_{18}(OCH_2)_3CCH_2OH]^{2-}$; experimental pattern is in black and simulated pattern is in blue.

6. Thermogravimetric analysis.

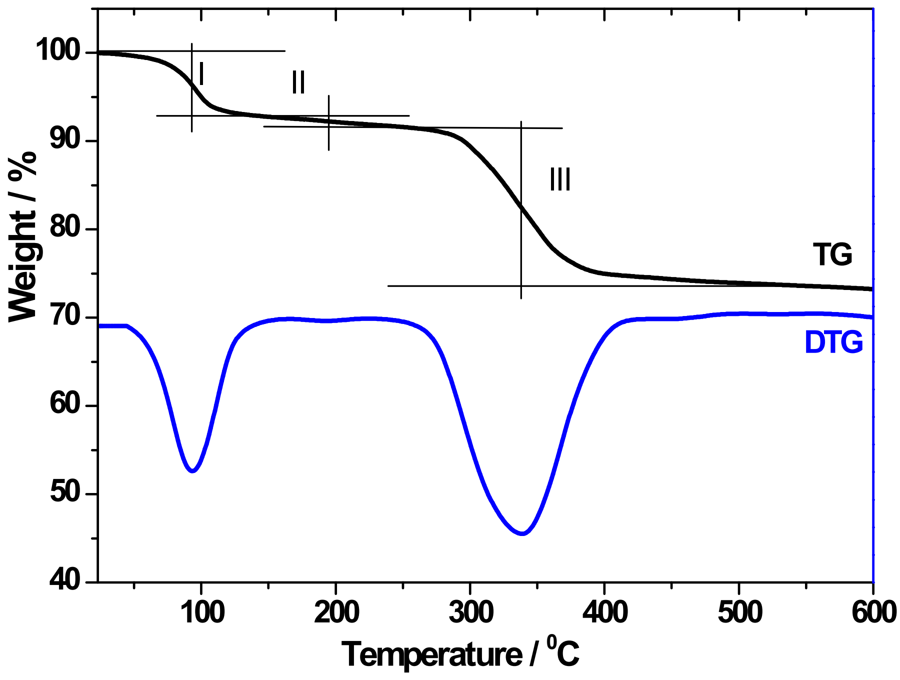

Fig. S6. Thermogram of $Na_2[TMA]_2[NiW_6O_{18}(OH)_3(OCH_2)_3CCH_2OH]\cdot 9H_2O$ (curves: TG — thermogravimetric curve, DTG — differential thermogravimetric curve).

Table 2. TGA results

Step	Temperature, °C	Mass-loss, %
I	33 – 160	7.28
II	160 – 230	0.81
III	230 – 530	18.12

References

1. G.M. Rozantsev, S.V. Radio, N.I. Gumerova, V.N. Baumer and O.B. Shishkin, *J. Struct. Chem.*, 2009, **50**, 296.
2. S.K. Roy and H.C. Mishra, *J. Indian Chem. Soc.,* 1978, **55**, 188.
3. Bruker SAINT V8.32B Copyright © 2005-2015 Bruker AXS
4. G.M. Sheldrick, 1996. *SADABS.* University of Göttingen, Germany.
5. G.M. Sheldrick, *Acta Cryst.,* 2008, **A64**, 112.
6. O.V. Dolomanov, L.J. Bourhis, R.J. Gildea, J.A.K. Howard and H. Puschmann, *J. Appl. Cryst.,* 2009, **42**, 339.
7. C.B. Huebschle, G.M. Sheldrick and B. Dittrich, *J. Appl. Cryst.*, 2011, **44**, 1281.
